# Space-Time Trends in Lassa Fever in Sierra Leone by ELISA Serostatus, 2012–2019

**DOI:** 10.3390/microorganisms9030586

**Published:** 2021-03-12

**Authors:** Jeffrey G. Shaffer, John S. Schieffelin, Mambu Momoh, Augustine Goba, Lansana Kanneh, Foday Alhasan, Michael Gbakie, Emily J. Engel, Nell G. Bond, Jessica N. Hartnett, Diana K. S. Nelson, Duane J. Bush, Matthew L. Boisen, Megan L. Heinrich, Megan M. Rowland, Luis M. Branco, Robert J. Samuels, Robert F. Garry, Donald S. Grant, the Viral Hemorrhagic Fever Consortium

**Affiliations:** 1 Department of Biostatistics and Data Science, School of Public Health and Tropical Medicine, Tulane University, New Orleans, LA 70112, USA; 2 Sections of Infectious Disease, Departments of Pediatrics and Internal Medicine, School of Medicine, Tulane University, New Orleans, LA 70112, USA; eengel@tulane.edu (E.J.E.); nbond@tulane.edu (N.G.B.); 3 Lassa Fever Program, Kenema Government Hospital, Kenema, Sierra Leone; mambumomoh@gmail.com (M.M.); augstgoba@yahoo.com (A.G.); lansanakanneh@gmail.com (L.K.); fodayalhasan37@gmail.com (F.A.); gbakiemichael@gmail.com (M.G.); robert.j.samuels@vanderbilt.edu (R.J.S.); 4 Department of Microbiology and Immunology, Tulane University, New Orleans, LA 70112, USA; jessica.n.hartnett@gmail.com (J.N.H.); rfgarry@tulane.edu (R.F.G.); 5 Zalgen Labs, LLC, Germantown, MD 20876, USA; dnelson@zalgenlabs.com (D.K.S.N.); dbush@zalgenlabs.com (D.J.B.); mboisen@zalgenlabs.com (M.L.B.); mheinrich@zalgenlabs.com (M.L.H.); mrowland@zalgenlabs.com (M.M.R.); lbranco@zalgenlabs.com (L.M.B.); 6 Vanderbilt Institute for Global Health, Vanderbilt University Medical Center, Nashville, TE 37203, USA

**Keywords:** Lassa fever, Lassa virus, case-fatality rate, enzyme-linked immunosorbent assay, Ebola virus disease, Sierra Leone

## Abstract

Lassa fever (LF) is a viral hemorrhagic disease found in Sub-Saharan Africa and is responsible for up to 300,000 cases and 5000 deaths annually. LF is highly endemic in Sierra Leone, particularly in its Eastern Province. Kenema Government Hospital (KGH) maintains one of only a few LF isolation facilities in the world with year-round diagnostic testing. Here we focus on space-time trends for LF occurring in Sierra Leone between 2012 and 2019 to provide a current account of LF in the wake of the 2014–2016 Ebola epidemic. Data were analyzed for 3277 suspected LF cases and classified as acute, recent, and non-LF or prior LF exposure using enzyme-linked immunosorbent assays (ELISAs). Presentation rates for acute, recent, and non-LF or prior LF exposure were 6.0% (195/3277), 25.6% (838/3277), and 68.4% (2244/3277), respectively. Among 2051 non-LF or prior LF exposures, 33.2% (682/2051) tested positive for convalescent LF exposure. The overall LF case-fatality rate (CFR) was 78.5% (106/135). Both clinical presentations and confirmed LF cases declined following the Ebola epidemic. These declines coincided with an increased duration between illness onset and clinical presentation, perhaps suggesting more severe disease or presentation at later stages of illness. Acute LF cases and their corresponding CFRs peaked during the dry season (November to April). Subjects with recent (but not acute) LF exposure were more likely to present during the rainy season (May to October) than the dry season (*p* < 0.001). The findings here suggest that LF remains endemic in Sierra Leone and that caseloads are likely to resume at levels observed prior to the Ebola epidemic. The results provide insight on the current epidemiological profile of LF in Sierra Leone to facilitate LF vaccine studies and accentuate the need for LF cohort studies and continued advancements in LF diagnostics.

## 1. Introduction

Lassa fever (LF) is an acute, viral, and often fatal illness that is endemic to Sierra Leone, Liberia, Guinea, and Nigeria [1]. More recently, LF has been observed in Mali, Ghana, and Benin [2]. LF was first described in Sierra Leone in the 1950s and was isolated in 1969 following the death of a nurse working at a hospital in Lassa, Nigeria [3,4,5]. The causal agent for LF is Lassa virus (LASV), an arenavirus found in Mastomys rats (*Mastomys natalensis*), a ubiquitous rodent in Sub-Saharan Africa [6,7]. LASV consists of up to seven lineages that are predominately localized in specific countries and generally cluster within geographic regions [8,9].

Between 300,000 and 500,000 LF cases resulting in 5000 deaths occur annually [10,11]. In parts of Sierra Leone and Liberia, it is estimated that 10–16% of admitted persons have LF infection [11]. Approximately 80% of people infected with LF are asymptomatic, while the remaining 20% of infections result in severe disease [2]. An estimated 80% of LF infections are acquired through contact with rodents, and 20% of infections are through human-to-human transmission via bodily fluids [12]. Persons at highest risk for LF reside in rural areas where Mastomys rats are prevalent [3]. Peak incidence for LF has been observed in the dry season for studies dating back to the 1980s [13]. However, recent studies suggest that that LASV survives better in humid conditions [14].

LF is classified as a National Institute of Allergy and Infectious Diseases (NIAID) Category A Biodefense Agent [15]. The standard therapy for LF is ribavirin, which is most efficacious within seven days of infection [16]. Symptomatic LF usually manifests through non-specific signs and symptoms (such as fever, headache, sore throat, muscle pain, chest pain, nausea, vomiting, diarrhea, cough, and abdominal pain), making differential diagnosis difficult with the presence of competing febrile illnesses such as malaria, typhoid fever, gastroenteritis, pneumonia, influenza, and many others [2,13,17]. Severe LF cases may manifest as facial swelling, fluid in the lung cavity, bleeding, and low blood pressure, and, in fatal cases, death usually occurs within 14 days of disease onset [2]. While there is currently no vaccine for LF, the largest initiative to date toward LF vaccine development was recently launched through the Coalition for Epidemic Preparedness Innovations (CEPI) global partnership [18].

The World Health Organization (WHO) estimates overall LF case-fatality rates (CFRs) at 1% [2], but those for hospitalized patients are usually considerably higher. In the early 1970s, CFRs for LF were 38% in hospitalized patients in Sierra Leone [19]. More recently, CFRs for LF have reached 69% in Sierra Leone and may exceed 80% in third-trimester pregnancies [18,20,21]. Other recent studies in Sierra Leone revealed LF CFRs of 67% in hospitalized patients and 63% in hospitalized children [22,23]. Countries reporting LF outside of Sierra Leone have historically reported lower CFRs. In 2020, a meta-analysis for 25 countries in Sub-Saharan Africa reported 29% CFRs for LF patients [24]. In Nigeria, CFRs declined from 94% in 2001 to 15% in 2018, while the caseload increased from 0.3 to 3.4% over the same period [25]. In several recent LF outbreaks in Nigeria, it was estimated that between 19.5% and 22.7% of confirmed cases died [26].

The highest incidence rates for LF have historically been observed in the Eastern Province of Sierra Leone [14]. Sierra Leone is primarily associated with the Lineage IV Josiah strain of LASV [27], which is the target of most vaccines [28]. The first LF studies in Sierra Leone were carried out from 1973 to 1992 but were halted from 1993–2001 during the Blood Diamonds War [29]. Since 2006, the Viral Hemorrhagic Fever Consortium (VHFC) has carried out research activities in Sierra Leone, focusing on building LF diagnostics capacity and implementing research studies on LF pathogenesis and its epidemiological risk factors [5,30,31,32].

Situated in the Eastern Province, Kenema District of Sierra Leone, Kenema Government Hospital (KGH) maintains the country’s sole LF isolation ward, and is one of only a few of its kind worldwide. Between 2014 and 2016, the largest recorded Ebola epidemic in history ravaged Sierra Leone, Liberia, and Guinea, resulting in over 11,000 deaths [33]. During this time, KGH served as the primary Ebola treatment facility in Sierra Leone and its LF ward was utilized to isolate Ebola patients [34]. The epidemic had a long-lasting and adverse impact on health care infrastructure where research and hospital operations struggled to continue [35,36]. Schieffelin et al. (2014) describe the effect of this epidemic and the clinical outcomes of Ebola patients presenting to KGH [37]. The detrimental impact on reproductive health services in Sierra Leone resulting from the epidemic was also recently described [38,39]. The WHO officially declared the end of the Ebola epidemic in Sierra Leone on 7 November 2015 [40].

A recent, large-scale study on the status and the space-time epidemiological profile of LF has yet to be carried out in the wake of the 2014–2016 Ebola epidemic. To address this gap, this study aimed to describe and characterize recent epidemiological and demographic factors for LF screenings at KGH, assess the impact of the 2014–2016 Ebola epidemic on LF clinical presentation patterns, and provide a current perspective about the status of LF in Sierra Leone to facilitate recent vaccine studies.

## 2. Materials and Methods

### 2.1. Study Site

Sierra Leone has a population of approximately 7.8 million people with a median age of 19.4 years, and about 57% of its people reside in rural areas [41,42]. The country is divided into four provinces and was formerly subdivided into 14 districts and 149 chiefdoms. A new geographical map was adopted in July 2017, dividing the country into four provinces, 16 districts, and 190 chiefdoms [43]. Seasonal rainfall in Sierra Leone occurs between May and October (referred to here as its “rainy season”), and low precipitation occurs between November and April (referred to here as its “dry season”) [44]. Situated in Kenema District, Eastern Province, Sierra Leone, KGH is a 350-bed regional hospital with a catchment area of approximately 670,000 persons [45]. KGH maintains the only LF treatment facility in Sierra Leone, including a 25-bed Lassa isolation ward and biosafety level-3 (BSL-3) laboratory, where up to 600 suspected LF cases are screened annually [17,20]. Suspected LF cases in Sierra Leone are initially evaluated at KGH or one of its peripheral health units (PHUs). Following clinical presentation, subjects meeting the LF suspected case definition provide blood samples for screening at the KGH Lassa Fever Laboratory. Subjects clinically diagnosed for LF are isolated and treated with ribavirin at the KGH Lassa Fever Ward. Subjects may self-present to KGH or may have their blood samples transported from PHUs to KGH for screening. Contact tracing is carried out for all confirmed LF cases by a dedicated surveillance team. In addition to providing screening services for residents of Sierra Leone, KGH occasionally screens samples for nearby health facilities in its neighboring country of Liberia.

### 2.2. Study Design and Inclusion Criteria

LF surveillance data were passively collected between 1 January 2012, and 31 December 2019, at KGH for symptomatic subjects meeting the suspected LF case definition, which covers three components of signs and symptoms: presence of fever, absence of signs of local inflammation, absence of clinical response to antimalarial treatment (or a broad-spectrum antibiotic), and a combination of at least two other clinical signs and symptoms known to be associated with LF [17]. The variables analyzed in this study included patient demographics (age at presentation, gender, and district of residence), date of clinical presentation, hospital admission status, date of illness onset, survival outcome, and LF seropositivity. Each of these variables was captured on the initial clinical presentation to KGH or one of its PHUs. The season of clinical presentation was determined according to the date of the first LF serostatus test, which usually occurred within 24 h of the blood draw. Patient survival outcomes were determined at hospital discharge unless subjects did not present to KGH, were transferred to another ward or clinic, or died before hospitalization. Inclusion criteria were residents of Sierra Leone with valid raw laboratory data for determining LF serostatus. It is worth mentioning that data captured during the 2014–2016 Ebola epidemic (particularly between May 2014 and January 2015) were often underreported or sporadically collected, and testing was frequently delayed due to the devastating impact on the KGH data and health information systems. 

### 2.3. Lassa Fever Clinical Database

The VHFC clinical database includes data sources captured since 2006 on referral, demographic, pre-admission, hospitalization, and laboratory outcomes. Data maintained in the database were collected and captured using a combination of paper-bound logbooks, case report forms (CRFs), medical charts, and electronic data files. Patient survival outcome was captured using enrollment logbooks, referral forms, and medical chart forms (and cross-verified over these sources to provide the best possible representation of survival outcome). The data capture process for LF screening at KGH is detailed by Shaffer et al. (2019) [46]. Data were abstracted from the VHFC clinical database for suspected LF cases between 1 January 2012, and 31 December 2019.

### 2.4. Lassa Fever Enzyme-Linked Immunosorbent Assay (ELISA) Diagnostics

LF serostatus was classified according to acute, recent (non-acute), and convalescent or prior LF exposure. Antigen (Ag)-capture ELISAs were used to detect LASV viremia through the presence of LASV nucleoprotein (NP) antigen (considered here as acute LF cases). Immunoglobin M (IgM)- and Immunoglobin G (IgG)-capture ELISAs were used to detect recent LF infection and convalescent LF exposure, respectively [30,31]. These immunoassays were developed on-site at KGH for diagnosis of LF [21]. The diagnostic sensitivity and specificity for the Ag ELISA were 94.1% and 83.7%, respectively [47].

While all subjects were tested for LF on clinical presentation, multiple tests were commonly performed to detect seroconversion or provide confirmatory results. For those cases where multiple testing occurred, the first test result was used to represent each observation for this study.

ELISA data were expressed as crude optical density (OD) values, standard deviations (SDs), dilutions, and positive and negative control values. The ELISAs were run in duplicate, and the replicate OD values were averaged and standardized by modeling average OD values against six concentration levels using a four-parameter logistic regression modeling approach [48]. An analog standardization approach was applied to the pool of negative control values. The cutoff values for determining seropositivity were defined as 2.5 SDs (for Ag serostatus) and 3 SDs (for IgM and IgG serostatuses) above the arithmetic mean of the standardized OD values. Values less than these cutoffs were considered as negative, and an indeterminate classification was not used.

### 2.5. Statistical Analysis

Data were expressed as frequencies and percentages or means and SDs as appropriate. Time periods preceding and following the 2014–2016 Ebola epidemic were based on the first and last reported Ebola cases at KGH (25 May 2014 and 7 November 2015, respectively). Pearson’s chi-square tests were used to assess differences in proportions among the comparison groups. Receiver operating characteristic curve (ROC) approaches were used to analyze a continuous age variable to determine its optimal breakpoint for determining survival outcome and acute LF infection. Data management and statistical analysis were carried out using the SAS System (version 9.4, SAS Institute, Inc., Cary, NC, USA). The type-I error threshold was set at 5%.

## 3. Results

### 3.1. Lassa Fever Suspected Cases by Year of Clinical Presentation

**Figure 1:**
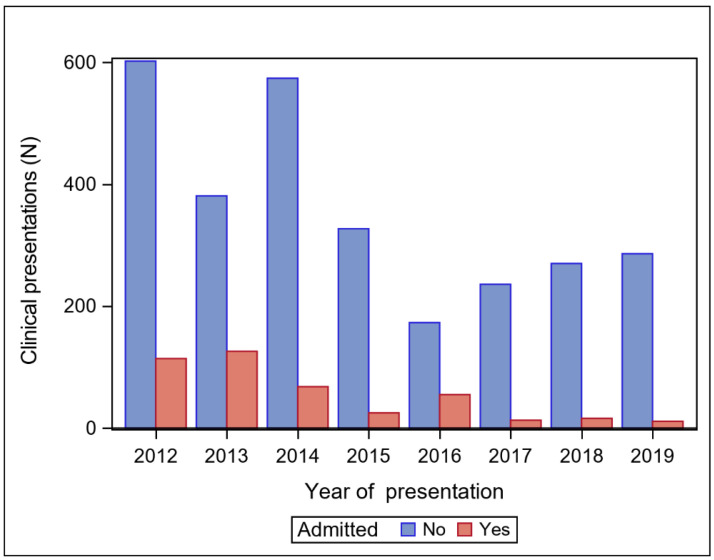
Lassa fever screenings at Kenema Government Hospital by admission status, 2012–2019. Note. Admitted refers to subjects admitted to KGH Lassa Fever Ward following confirmed Lassa fever diagnosis.

Data were analyzed for *n* = 3277 suspected LF cases screened at the KGH LF Laboratory between 1 January 2012 and 31 December 2019. A total of 428 subjects were hospitalized at KGH, and the remaining 2849 subjects were not admitted (Figure 1).

Clinical presentations and screenings for LF were skewed toward the beginning of the time frame, peaking in 2012 (716 screenings) and were lowest in 2016 (228 screenings). Hospital admissions peaked in 2013 (126 admissions) and were lowest in 2019 (11 admissions). The proportion of hospital admissions to clinical presentations peaked in 2013 and 2016 (24.9 and 24.1 admissions per presentation, respectively). Between 2016 and 2019, there was a 30.2% increase in suspected LF presentations (228 presentations versus 297 presentations in 2016 and 2019, respectively).

### 3.2. Serostatus Group Comparisons

The *n* = 3277 suspected LF cases were classified in terms of Ag and IgM ELISA serostatus groups as Ag+ (acute LF exposure); Ag−/IgM+ (recent, non-acute LF exposure); and Ag−/IgM− (non-LF or prior LF exposure). The baseline characteristics for the serostatus groups are shown in Table 1.

The serostatus groups differed according to hospital admission rate, age distribution, survival outcome, duration between illness onset and clinical presentation, IgG serostatus, and season of clinical presentation. Six percent (195/3277) of samples tested positive for acute LF exposure (Ag+). Among these samples, 54% (106/195) were for subjects admitted to KGH, while 46% (89/195) were for non-admitted subjects. Those subjects in the Ag+ group not admitted to the hospital were commonly due to deaths occurring prior to arrival to KGH or serostatus changes due to follow-up testing.

Among all tested samples, 31.5% (1033/3277) showed evidence of acute or recent LF infection, while the majority of samples (68.5% [2244/3277]) did not reveal any evidence of acute or recent LF exposure. Survival outcomes were available for 135 of the 195 Ag+ subjects, and the overall CFR was 78.5% (106/135). Serostatus results differed by age category, where subjects aged over 40 years were more commonly observed in the Ag−/IgM+ or Ag−/IgM− groups (19.7% [307/1552] and 17.7% [117/661] for the Ag−/IgM+ and Ag−/IgM− groups, respectively versus 9.0% [17/188] for the Ag+ group; *p* < 0.001). Interestingly, IgG positivity was more likely to occur in subjects aged under 15 years than those aged at least 15 years (Table S1, *p* < 0.001).

Ag−/IgM− subjects were more likely than the other two serostatus groups to present to a health facility within seven days following illness onset (66.0% [357/541] for the Ag−/IgM− group versus 43.0% [47/109] and 43.0% [145/337] for the Ag+ Ag−/IgM+ groups, respectively; *p* < 0.001). The Ag−/IgM+ group was significantly more likely to present during the rainy season than the other two comparison groups (61.4% [513/836] for the Ag−/IgM+ group versus 43.6% [85/195] and 47.9% [1067/2229] for the Ag+ and Ag−/IgM− groups, respectively; *p* < 0.001). Among the 2244 Ag−/IgM− subjects, 2051 were tested for convalescent LF exposure (IgG positivity), and IgG serostatus was positive for 33.2% (682/2051) of these subjects.

### 3.3. Fatality Rates by Serostatus

**Figure 2:**
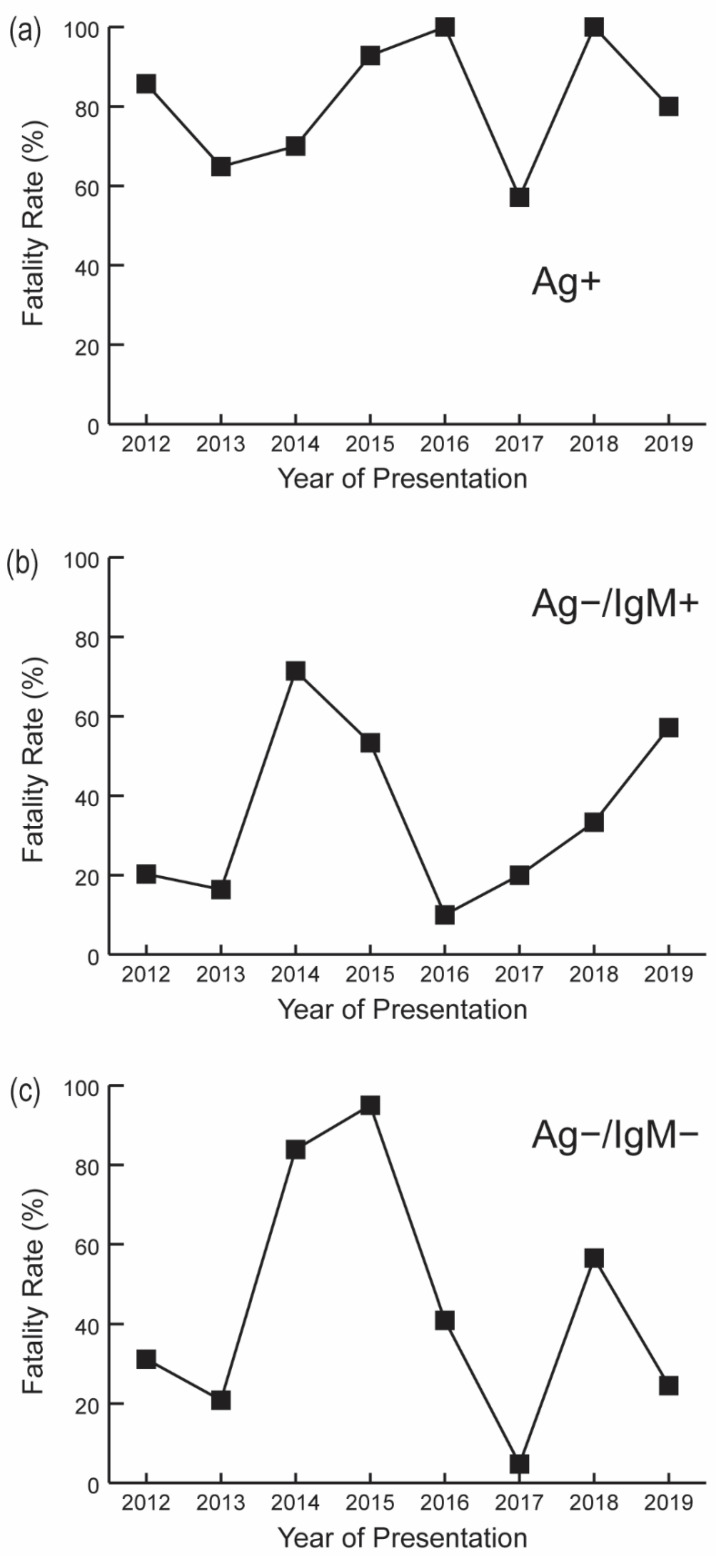
Annual fatality rates by Lassa fever serostatus. (**a**) Acute LF exposure; (**b**) Recent LF exposure (Ag−/IgM+); (**c**) Non-LF or prior LF exposure (Ag−/IgM−).

Fatality rates were plotted by serostatus and year of clinical presentation (Figure 2).

CFRs among Ag+ cases ranged from 57.1% (8/14) in 2017 to 100.0% (8/8) in 2018. Fatality rates for Ag−/IgM+ subjects ranged from 10.0% (1/10) in 2016 to 71.4% (25/35) in 2014. Ag−/IgM− fatality rates peaked in 2015 and were lowest in 2017 (95.0% [38/40] versus 4.8% [2/42], respectively). Average CFRs prior to the 2014–2016 Ebola epidemic (2012–2013) were 73.8% (48/65) compared with 88.9% (16/18) following the epidemic (2018–2019). 

CFRs for the Ag+ group were significantly higher in the dry season (86.1% [62/72]) than the rainy season (69.8% [44/63]; *p* = 0.022). Patient survival outcome among Ag+ subjects was not significantly associated with hospital admission status, gender, age, district of residence, duration between illness onset and clinical presentation, or IgG serostatus (Table S2). However, admission status for the Ag−/IgM+ and Ag−/IgM− groups coincided with an increased likelihood of survival (Table S3, *p* < 0.001).

IgG seropositivity was not associated with CFRs for the Ag+ or Ag−/IgM+ groups (Table S1). However, IgG serostatus was associated with the CFRs in Ag−/IgM− subjects, where increased IgG positivity was coincident with increased CFRs (*p* < 0.001). Please note, however, that this result should be interpreted with caution as survival outcome data were sparse for the Ag−/IgM− group as subjects in this group were not usually admitted to the hospital.

### 3.4. Breakpoints for Continuous Age Predictor

**Figure 3:**
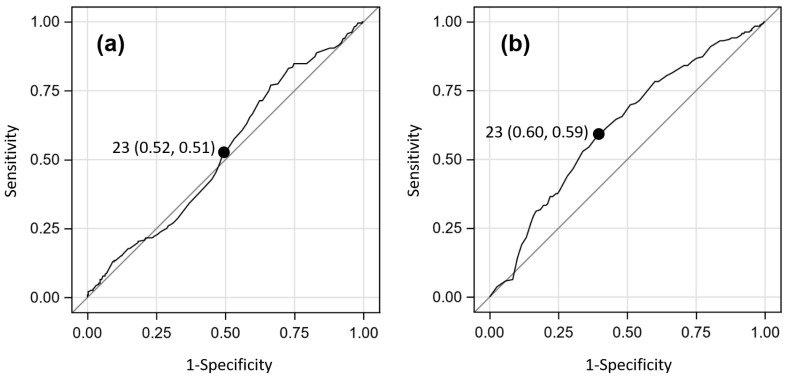
Receiver Operating Characteristic (ROC) analyses for subject age at clinical presentation for suspected Lassa fever cases. (**a**) ROC results for determining patient survival outcome on clinical presentation; (**b**) ROC result for determining acute Lassa fever seropositivity on clinical presentation. The leftmost numbers inside the plot are the age values, and the middle and rightmost values correspond to their respective sensitivity and 1-specificity values.

As subject age was previously classified categorically (<5, 5–14, 15–40, >40 years), the optimal age breakpoint that would determine patient survival outcome and acute LF seropositivity status was evaluated. Figure 3 shows the ROC analyses for age value breakpoints with respect to predicting patient survival outcome and antigen seropositivity. 

A breakpoint of 23 years best classified these data according to patient survival outcome (sensitivity = 0.52, specificity = 0.51, area under the curve [AUC] = 0.525; *p* = 0.004). The analyses for acute LF seropositivity yielded a higher AUC value, which also occurred at age = 23 years (sensitivity = 0.60, specificity = 0.59, AUC = 0.617; *p* = 0.005). Together, these results suggest that an age of 23 years at clinical presentation is an influential breakpoint in determining both patient survival and acute LF seropositivity.

### 3.5. Space-Time Trends in Suspected LF Presentations and Confirmed Cases

**Figure 4:**
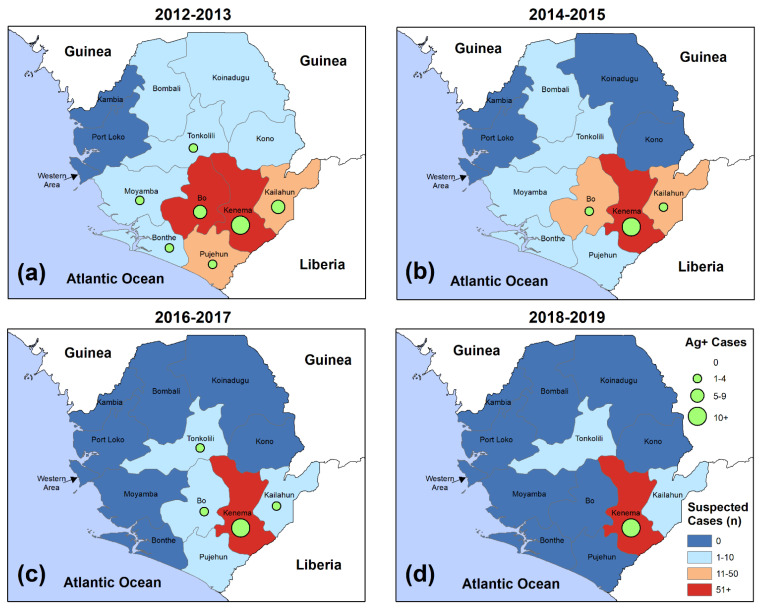
Suspected and confirmed Lassa fever cases screened at Kenema Government Hospital, 2012–2019. (**a**) Presentations between 1 January 2012 and 31 December 2013; (**b**) Presentations between 1 January 2014 and 31 December 2014; (**c**) Presentations between 1 January 2016 and 31 December 2017; (**d**) Presentations between 1 January 2018 and 31 December 2019. Following the 2014–2016 Ebola epidemic, suspected and confirmed cases were observed in closer proximity to Kenema District.

Spatiotemporal patterns were mapped to illustrate LF clinical presentations and confirmed cases by two-year time intervals (Figure 4).

Study subjects primarily resided in the hyperendemic Kenema District or one of its neighboring districts (Bo, Kailahun, or Pujehun). Both screenings and confirmed cases were increasingly clustered over time in the Kenema region. From 2012 to 2013, Kenema District accounted for 69.7% (515/739) of all screenings, while this district accounted for 97.9% (139/142) of screenings from 2018 to 2019. From 2012 to 2013, seven of the 14 districts (50%) included at least one acute case. Between 2018 and 2019, 100.0% (13/13) of the Ag+ cases resided in Kenema District.

### 3.6. Seasonality of Lassa Fever Presentations

Table 2 shows patient characteristics by season of clinical presentation. Acute LF cases (Ag+) were more likely to present during the dry season than the rainy season (6.9% [110/1595] of presentations occurred during the dry season versus 5.1% [85/1665] during the rainy season; *p* = 0.031). Those subjects with recent or prior LF infection (Ag−/IgM+) were more likely to present in the rainy season than the dry season (33.6% [559/1665] in the rainy season versus 22.3% [356/1595] in the dry season; *p* < 0.001). Age at clinical presentation differed between the rainy and dry seasons, particularly in subjects aged under five years (*p* = 0.005). Subjects aged under five years represented 14.8% (177/1200) of the presenting population in the rainy season versus 10.1% (121/1195) in the dry season. IgG serostatus did not differ by season of clinical presentation (33.9% [541/1598] in the rainy season versus 33.3% [467/1401] in the dry season; *p* = 0.763).

**Figure 5:**
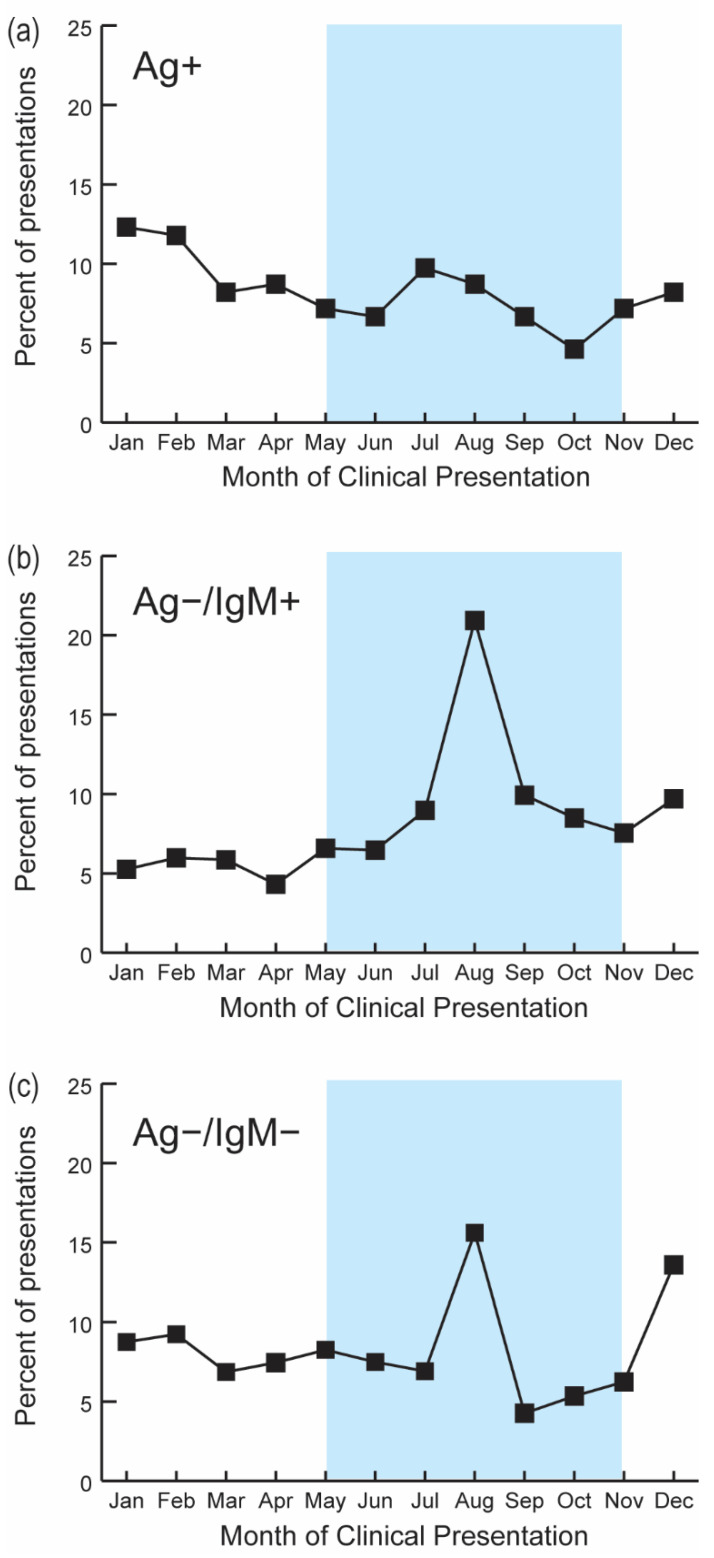
Monthly distribution of suspected LF cases by serostatus. Month of clinical presentation was determined according to the date the initial ELISA Ag and IgM tests were performed. (**a**) Acute Lassa fever exposure (Ag+), (**b**) Recent Lassa fever exposure (Ag−/IgM+); (**c**) Non-Lassa fever cases or prior Lassa fever exposure (Ag−/IgM−). All tests were for suspected LF cases defined according to its suspected case definition. The blue-shaded regions represent the seasonal rainfall period in Sierra Leone (May to October).

LF-related observations peaked at the beginning of the rainy season (1665 presentations in the rainy season versus 1595 presentations in the dry season), while acute cases peaked in January (23 acute cases) and February (24 acute cases; Figure 5).

Interestingly, recent LF exposures (Ag−/IgM+) most frequently presented in August (at the middle of the rainy season) with 21% [175/836] of the Ag−/IgM+ presentations. Non-LF or prior LF subjects (Ag−/IgM−) also most frequently presented in the rainy season, accounting for 16% (348/2229) of non-LF subjects in August. The spatial distribution of presentations and confirmed cases did not differ between the rainy and dry seasons (*p* = 0.335; Figure S1).

### 3.7. Impact of the 2014–2016 Ebola Epidemic on LF Epidemiological Factors

Temporal breakpoints for classifying the study data according to periods preceding, during, and following the 2014–2016 Ebola epidemic were determined according to the first and last reported Ebola cases in Sierra Leone (25 May 2014 and 7 November 2015, respectively). Table 3 shows a comparison of the patient characteristics according to the three time periods. LF screenings during the Ebola period were irregular at KGH between 25, May, 2014, to 17, November, 2014, due to the unprecedented impact of the epidemic on isolation and testing capacity and data systems.

Following the Ebola epidemic, subjects were increasingly likely reside in Kenema District (accounting for 96.0% [288/300] of all suspected cases following the epidemic versus 69.1% [569/824] of all cases prior to the epidemic, *p* < 0.001). Other factors that differed between the pre-and post-Ebola time periods were: Hospitalizations (22.1% [306/1384] versus 8.8% [98/1012], *p* < 0.001); percentage of presenting females (59.0% [811/1375] versus 54.7% [583/1065], *p* = 0.036); Ag−/IgM+ serostatus (36.4% [504/1384] versus 16.5% [183/1110], *p* < 0.001); percentage of IgG+ subjects (21.6% [295/1365] versus 43.0% [378/879], *p* < 0.001); and percentage of presentations during the rainy season (55.4% [767/1384] versus 39.0% [428/1097], *p* < 0.001). Overall fatality rates did not significantly differ between the pre- and post-Ebola time periods (39.8% [86/216] following the epidemic versus 33.5% [131/391] before the epidemic; *p* = 0.120).

## 4. Discussion

We observed distinct spatiotemporal patterns in LF presentations and confirmed cases in Sierra Leone between 2012 and 2019. Both suspected and confirmed LF cases primarily resided in Kenema or one of its neighboring districts, which is at least partially attributable to self-presentation bias. Additionally, the testing occurred in Kenema, which is Sierra Leone’s third-largest city and provides easier access for those living near the testing site than those residing in more rural parts of the country. The known frequent travel between Kenema and Liberia may also contribute to the higher observed case frequencies observed in Kenema. However, over 30% of the Ag−/IgM− group tested positive for convalescent LF exposure, which occurred both inside and outside of Kenema District. This finding suggests that LF remains highly prevalent across Sierra Leone.

It is estimated that about 80% of people who become infected with LASV are asymptomatic and that 1 in 5 infections result in severe disease [2]. The sample of LF cases here was clearly a subset of those with severe, symptomatic disease, but data were not collected for asymptomatic subjects. For this reason, the passive case detection approach applied undoubtedly underestimates (and likely substantially underestimates) the actual number of LF cases.

### 4.1. Testing Is Likely Prioritizing Subjects at Late Stages of Acute LF Infection

There is a direct relationship between CFR temporal fluctuations with screening activity. LF CFRs in Sierra Leone remain higher than those reported in other LF endemic countries. The high CFRs observed in this study suggest that current testing approaches may capture only a small portion of highly symptomatic cases. This observation is supported by the findings in this study as the majority of LF cases presented at or after seven days following illness onset. Since ribavirin therapy is most efficacious within seven days following disease onset, reaching patients at earlier stages of the illness would reduce CFRs. In 2019, Nigeria experienced the largest LF outbreak to date, while the country’s CFRs declined in 2020, partly due to enhanced screening [26].

Notably, fatality rates among subjects without acute or recent exposure were remarkably high (54%). It is worth mentioning, however, that survival outcome data was often unavailable for these subjects as they were not typically admitted to the KGH Lassa Fever Ward. Nonetheless, the majority of Ag−/IgM− subjects with observed survival outcomes died of an unknown febrile illness, which accentuates the illness severity in the presenting population. While differential diagnoses were not included in this work, the most likely contributing illness is malaria as it remains highly endemic across Sierra Leone. In an earlier study in Sierra Leone, over half of non-LF or prior LF subjects had detectable levels of *Plasmodium falciparum* [20].

Among the Ag−/IgM+ and Ag−/IgM− serostatus groups, subjects admitted to KGH experienced lower fatality rates compared with non-admits, suggesting that hospital care at the KGH Lassa Ward is effective in reducing mortality in these groups. It was beyond the scope of this study to evaluate the efficacy of ribavirin therapy, but it is worth mentioning that the ELISA diagnostic test results constitute only part of the LF confirmation process by the clinicians.

A particular challenge lies in triaging suspected LF patients at rural PHUs. Indeed the majority of the subjects for this study did not test positive for acute or recent LF exposure and ideally would be screened at facilities outside of KGH. However, screening practices necessitated the transport of a blood sample from the PHUs to KGH due to the limited supply of rapid LF diagnostics. Ideally, screening would occur on a rapid basis to meet the seven-day threshold for ribavirin efficacy. Another consideration for improved triaging centers on reevaluating suspected case definitions for LF and competing febrile illnesses. Specific, measurable signs and symptoms are desirable but must consider facilities that may lack medical equipment needed for their implementation.

### 4.2. The Definition of Confirmed LF Cases Significantly Influences CFRs

Differential LF diagnosis for this study focused on acute (Ag+) exposure. Recent, non-acute LF exposure (Ag−/IgM+) does, however, occasionally result in hospitalization and confirmed LF diagnoses. Reframing the LF case definition in terms of acute or recent exposures would have considerably reduced the CFRs reported here. More specifically, the overall CFR of 79% for acute cases was significantly higher than the fatality rate for recent LF exposures (31%). The combined fatality rate for acute or recent exposures was 49%. However, in our experience, recent LF exposures (Ag−/IgM+) are often consistent with LF recovery and do not regularly warrant hospitalization and thus were not considered in determining the CFRs. Interestingly, fatality rates among recent exposures (Ag−/IgM+) were lower than those for convalescent exposures (IgG+). A possible explanation for this occurrence is that some Ag−/IgM+ subjects may be skewed toward more acute exposure stages of LF. Additionally, many of the Ag−/IgM− subjects here may have been at severe stages of competing, unknown febrile illnesses.

### 4.3. LF Remains Endemic in Sierra Leone

Among non-LF or prior LF exposures (Ag−/IgM−), over 30% of subjects tested positive for convalescent exposure (IgG+). This finding was observed for subjects residing both inside and outside of Kenema District, suggesting that LF infection remains highly endemic to Sierra Leone. The likelihood of subjects with convalescent exposure previously experiencing severe symptoms was beyond the scope of this study. IgG seropositivity did not appear to impact CFRs in Ag+ or Ag−/IgM+ subjects. Convalescent LF subjects were more likely to have fatal outcomes (compared with non-LF subjects) in the Ag−/IgM− group, but the outcome data were sparse in Ag−/IgM− subjects which may impact its interpretation.

### 4.4. LF Case-Fatality Rates Are Higher in the Dry Season and Recent, Non-Acute LF Exposures Are More Likely to Present in the Rainy Season

Peak LF incidence occurred during the dry season, which was consistent with previous studies. Additionally, we found that CFRs were higher during the dry season (compared with the rainy season) among acute LF cases. Seasonal trends differed by stage of illness, where recent, non-acute exposures (Ag−/IgM+) were significantly more likely to present during the rainy season than the other serostatus groups. We observed an increasing trend in the proportion of Ag−/IgM+ observations during the rainy season, suggesting that those with recent LF infection, even without severe hemorrhagic manifestations, may be predisposed to other febrile illnesses that were impacted by factors related to precipitation such as malaria.

### 4.5. Suspected LF Case Frequencies Have Yet to Recover Following the 2014–2016 Ebola Epidemic

The impact of the 2014–2016 Ebola epidemic on the health information systems in Sierra Leone was partially quantified in this study. We observed increased duration between illness onset and clinical presentation and sharp declines in self-presentations of symptomatic cases (and fewer LF cases overall) after the epidemic. While these findings are partly attributable to the reorganization of screening locations in Sierra Leone following the epidemic, the utilization of health care services following the epidemic has also generally declined for health outcomes in other parts of West Africa [49,50,51,52]. Declines in suspected LF presentations this study coincided with higher CFRs. Together, these findings suggest that either symptomatic subjects are delaying presentation following illness onset or that there have been actual changes in illness severity. The extremely high CFRs following the epidemic are likely due to clinical presentations at later stages of illness. It is therefore likely that suspected LF case presentations and CFRs will ultimately return to their pre-Ebola levels. We refrained from drawing conclusions about patterns observed during the Ebola period as LF testing was not routinely carried out over that time period. It is also possible that some patients with LF may have presented as suspected Ebola patients during the Ebola time period.

### 4.6. Only a Few Confirmed Cases LF Cases Are Needed to Constitute an LF Outbreak

In terms of what constitutes an outbreak for LF, every confirmed case in Sierra Leone triggers a case investigation by the KGH outreach team that involves interviewing and training household contacts about LF. Therefore, each case observed at KGH is essentially considered and treated as though it were an outbreak. It is worth mentioning that the number of beds for LF at the KGH isolation Ward is limited. Thus, even a few isolated cases of LF can place an extreme burden on health systems and contact tracing personnel. A recent LF outbreak occurred in Nigeria, resulting in 365 cases and 114 deaths, overwhelmed its only dedicated LF ward at Irrua Specialist Teaching Hospital in Edo state with just 24 beds [53].

### 4.7. Subjects Aged over 40 Years Are Less Frequently Observed in the Acute or Recent Serostatus Groups

Interestingly, serostatus results differed by age category, where subjects aged over 40 years were more commonly observed in the Ag−/IgM− group than the Ag+ or Ag−/IgM+ groups. This finding could potentially be due to less LF exposure experienced in older age groups or increased immunity in older populations. Increased immunity in older age populations is at least partially supported by our finding that IgG positivity was more likely to occur in subjects aged at least 15 years (Table S1). There may be a relationship between convalescent exposure in older adults and those found in recent vaccine studies, where older adults experienced fewer side effects than younger adults [54].

### 4.8. Limitations

Perhaps the most significant limitation for this study was self-presentation bias, which precluded causal inference. The majority of study subjects resided in Kenema District in each year of the study. Further, data systems were overwhelmed during the 2014–2016 Ebola epidemic, which often led to incomplete or lagged reporting during that period. The data captured here did not include survival outcome data for most of the non-hospitalized subjects. Survival outcomes for hospitalized subjects were captured at discharge, so the reported CFRs may underestimate mortality as some LF cases may have died following hospital discharge. Test results were determined according to the first test on the first blood draw, and repeat testing often occurred (resulting in different serostatus results), which may explain some of the non-admissions and admissions in the Ag+ and Ag−/IgM− serostatus groups, respectively. It is also possible that some LF cases initially presented to a PHU while not completing follow-up recommendations following antimalarial or general antibiotic therapy (one of the first steps in meeting the suspected LF case definition), which may further explain some of the non-admissions classified with acute LF exposure. The rainy and dry seasons were classified according to historical rainfall patterns (May–October and November–April), but these patterns are known to fluctuate on an annual basis. The ELISA diagnostics detected Lineage IV LASV strains, and the Ag−/IgM− group may have included subjects experiencing symptoms from other LASV strains.

## 5. Conclusions

The 2014–2016 Ebola epidemic and increased globalization have raised awareness about the potential global threat of emerging infectious diseases such as Ebola virus disease and LF. The extremely high CFRs observed in this study suggest that more mobile and rapid diagnostic testing for LF is critically needed across Sierra Leone. While the number of confirmed LF cases is currently relatively low, they coincided with higher CFRs than before the Ebola epidemic. It is therefore likely that the LF case and presentation loads in Sierra Leone will resume to levels observed before the Ebola epidemic. Studies characterizing the epidemiological profiles for LF should remain a priority at local levels to detect emerging outbreaks, monitor and evaluate diagnostic testing strategies, and facilitate LF vaccine studies.

## Figures and Tables

**Table 1:** Characteristics of Lassa fever screenings by serostatus, Kenema Government Hospital, 2012–2019. Note. All results expressed as frequencies and percentages unless indicated otherwise. Those characteristics with aggregate frequencies less than their respective aggregate serostatus group sample sizes reflect missing characteristic responses. Ag+ = Samples testing positive according to Ag ELISA (acute Lassa exposure); Ag−/IgM+ = Samples testing negative according to Ag ELISA and positive according to IgM ELISA (recent Lassa exposure); Ag−/IgM− = Samples testing negative according to both Ag and IgM ELISA.

Characteristic	Serostatus			*p* Value ^d^
	Ag+ (*n* = 195)	Ag−/IgM+ (*n* = 838)	Ag−/IgM− (*n* = 2244)	
Admission status				
Admitted	106 (54)	159 (19)	163 (7)	<0.001
Not admitted	89 (46)	679 (81)	2081 (93)	
Gender				
Female	101 (52)	435 (57)	1071 (56)	0.471
Male	92 (48)	326 (43)	850 (44)	
Age in years				
<5	35 (19)	84 (13)	180 (12)	<0.001
5–14	33 (17)	97 (14)	175 (11)	
15–40	103 (55)	363 (55)	890 (57)	
>40	17 (9)	117 (18)	307 (20)	
District of residence				
Bo	12 (9)	42 (12)	79 (12)	0.712
Kenema	104 (79)	265 (75)	506 (77)	
Other	16 (12)	47 (13)	72 (11)	
Survival outcome ^a^				
Died	106 (79)	67 (31)	259 (54)	<0.001
Discharged	29 (21)	149 (69)	222 (46)	
Time since illness onset				
<7 days	47 (43)	145 (43)	357 (66)	<0.001
≥7 days	62 (57)	192 (57)	184 (34)	
IgG serostatus ^b^				
Positive	42 (23)	291 (37)	682 (33)	0.001
Negative	140 (77)	492 (63)	1369 (67)	
Season of presentation ^c^				
Dry	110 (56)	323 (39)	1162 (52)	<0.001
Rainy	85 (44)	513 (61)	1067 (48)	

^a^ Patient survival outcome was determined at hospital discharge (or following initial consultation for subjects not admitted to KGH). ^b^ IgG serostatus = Immunoglobin G ELISA test result for detecting convalescent Lassa exposure. ^c^ Rainy and dry seasons were defined as 1 May–31 October and 1 November–30 April, respectively. ^d^ Calculated using Pearson’s chi-square test assessing general differences in proportions between serostatus groups.

**Table 2:** Characteristics of suspected Lassa fever screenings by season of clinical presentation, Kenema Government Hospital, 2012–2019. Note. All results expressed as frequencies and percentages unless indicated otherwise. Those characteristics with aggregate frequencies less than their respective aggregate serostatus group sample sizes reflect missing characteristic data.

Characteristic	Season of Presentation ^e^		*p* Value ^f^
	Rainy (*n* = 1665)	Dry (*n* = 1595)	
Admission status			
Admitted	209 (13)	219 (14)	0.320
Not admitted	1456 (87)	1376 (86)	
Gender			
Female	769 (58)	834 (54)	0.073
Male	562 (42)	698 (46)	
Age, years			
<5	177 (15)	121 (10)	0.005
5–14	141 (12)	163 (14)	
15–40	663 (55)	691 (58)	
>40	219 (18)	220 (18)	
District of residence			
Bo	70 (12)	63 (11)	0.335
Kenema	427 (77)	446 (76)	
Other	59 (11)	76 (13)	
Survival outcome ^a^			
Died	136 (42)	295 (58)	<0.001
Discharged	187 (58)	212 (42)	
Time since illness onset			
<7 days	267 (54)	280 (57)	0.347
≥7 days	227 (46)	211 (43)	
Ag serostatus ^b^			
Positive	85 (5)	110 (7)	0.031
Negative	1580 (95)	1485 (93)	
IgM serostatus ^c^			
Positive	559 (34)	356 (22)	<0.001
Negative	1106 (66)	1239 (78)	
IgG serostatus ^d^			
Positive	541 (34)	467 (33)	0.763
Negative	1057 (66)	934 (67)	

^a^ Patient survival outcome was determined at hospital discharge (or following initial consultation for subjects not admitted to KGH). ^b^ Ag serostatus = Antigen ELISA test result for detecting acute Lassa fever exposure. ^c^ IgM serostatus = Immunoglobin M ELISA test result for detecting recent Lassa fever exposure. ^d^ IgG serostatus = Immunoglobin G ELISA test result for detecting convalescent Lassa fever exposure. ^e^ Rainy and dry seasons were defined as 1 May–31 October and 1 November–30 April, respectively. ^f^ Calculated using Pearson’s chi-square test assessing general differences in proportions between serostatus groups.

**Table 3:** Characteristics of suspected Lassa fever cases before, during, and after the 2014–2016 Ebola epidemic, Kenema Government Hospital, 2012–2019. Note. All results expressed as frequencies and percentages unless indicated otherwise. Those characteristics with aggregate frequencies less than their respective aggregate serostatus group sample sizes reflect missing characteristic responses. Date classifications for comparisons between the pre- and post-Ebola periods were based on the first and last reported cases at KGH (25 May 2014 and 7 November 2015, respectively).

Characteristic	Time Period			*p* Value ^f^
	Pre-Ebola *n* = 1384	Ebola ^e^ *n* = 783	Post-Ebola *n* = 1110	
Admission status				
Admitted	306 (22)	24 (3)	98 (9)	<0.001
Not admitted	1078 (78)	759 (97)	1012 (91)	
Gender				
Female	811 (59)	213 (49)	583 (55)	0.036
Male	564 (41)	222 (51)	482 (45)	
Age, years				
<5	158 (13)	17 (7)	124 (13)	0.346
5–14	171 (14)	27 (11)	107 (11)	
15–40	676 (56)	141 (57)	539 (58)	
>40	212 (17)	61 (25)	168 (18)	
District				
Bo	131 (16)	0 (0)	2 (1)	<0.001
Kenema	569 (69)	18 (95)	288 (96)	
Other	124 (15)	1 (5)	10 (3)	
Survival outcome ^a^				
Died	131 (34)	215 (96)	86 (40)	0.120
Discharged	260 (66)	10 (4)	130 (60)	
Serostatus ^b^				
Ag+	85 (6)	21 (3)	89 (8)	<0.001
Ag−/IgM+	504 (36)	151 (19)	183 (16)	
Ag−/IgM−	795 (58)	611 (78)	838 (76)	
Time since illness onset				
<7 days	314 (46)	7 (44)	228 (80)	<0.001
≥7 days	373 (54)	9 (56)	56 (20)	
IgG serostatus ^c^				
Positive	295 (22)	342 (44)	378 (43)	<0.001
Negative	1070 (78)	430 (56)	501 (57)	
Season of presentation ^d^				
Rainy	767 (55)	470 (60)	428 (39)	<0.001
Dry	617 (45)	309 (40)	669 (61)	

^a^ Patient survival outcome was determined at hospital discharge (or following initial consultation for subjects not admitted to KGH). ^b^ Ag+ = Samples testing positive according to Ag ELISA (acute Lassa fever exposure); Ag−/IgM+ = Samples testing negative according to Ag ELISA and positive according to IgM ELISA (recent Lassa exposure); Ag−/IgM− = samples testing negative according to both Ag and IgM ELISA. ^c^ IgG serostatus = Immunoglobin G ELISA test result for detecting convalescent Lassa fever exposure. ^d^ Rainy and dry seasons were defined as 1 May–31 October and 1 November–30 April, respectively. ^e^ LF screenings over this period were skewed as suspected LF cases were not regularly seen or tested at KGH between 25 May 2014 to 17 November 2014. ^f^ Calculated using Pearson’s chi-square test comparing pre- and post-Ebola time periods.

## Data Availability

Data are contained within the article or supplementary material. The data presented in this study are available in Table 1, Table 2 and Table 3 and Tables S1–S3.

## Supplementary Materials

The following are available online at https://www.mdpi.com/2076-2607/9/3/586/s1, Table S1: IgG serostatus by age group, Kenema Government Hospital Lassa Fever Ward, 2012–2019, Table S2: Characteristics of confirmed Lassa fever cases by survival outcome, Kenema Government Hospital Lassa Fever Ward, 2012–2019, Table S3: Hospital admission status by survival outcome, Kenema Government Hospital Lassa Fever Ward, 2012–2019, Figure S1: Spatial distribution of Lassa fever screenings and confirmed LF cases at Kenema Government Hospital by season of clinical presentation, 2012–2019.
