# Supplementary material for: Space-Time Trends in Lassa Fever in Sierra Leone by ELISA Serostatus, 2012–2019
Source: Microorganisms. 2021 Mar 12;9(3):586. doi: 10.3390/microorganisms9030586 (PMC8000031; doi:10.3390/microorganisms9030586)
Supplement: Supplementary file 1 [file microorganisms-09-00586-s001.pdf]

Supplemental Information

Characteristic	IgG serostatus[e]		p value[f]
	IgG+ (n = 1,015)	IgG− (n = 2,001)	
Admission status			
Admitted	96 (9)	291 (15)	.001
Not admitted	919 (91)	1,710 (85)	
Gender			
Female	473 (56)	994 (56)	.969
Male	369 (44)	778 (44)	
District			
Bo	13 (5)	114 (14)	<.001
Kenema	213 (86)	591 (73)	
Other	23 (9)	107 (13)	
Time since illness onset			
< 7 days	128 (55)	369 (54)	.779
≥ 7 days	103 (45)	310 (46)	
Season of presentation[a]			
Rainy	541 (54)	1,057 (53)	.762
Dry	467 (46)	934 (47)	
Age, years			
< 5	33 (5)	225 (15)	<.001
5-14	58 (9)	213 (14)	
15-40	399 (62)	819 (55)	
> 40	153 (24)	241 (16)	
Ag+[b]			
Died	12 (63)	85 (80)	.101
Discharged	7 (37)	21 (20)	
Ag−/IgM+[c]			
Died	24 (34)	40 (31)	.634
Discharged	47 (66)	91 (69)	
Ag−/IgM−[d]			
Died	111 (68)	142 (48)	<.001
Discharged	52 (32)	156 (52)	

Table S1. IgG serostatus by age group, Kenema Government Hospital Lassa Fever Ward, 2012-2019. Note. All results expressed as frequencies and percentages unless indicated otherwise. Those characteristics with aggregate frequencies less than their respective aggregate serostatus group sample sizes reflect missing characteristic data.

[a] Rainy and dry seasons were defined as May 1-October 31 and November 1-April 30, respectively.
[b] Ag+ = Samples testing positive according to Ag ELISA (acute Lassa exposure).
[c] Ag−/IgM+ = Samples testing negative according to Ag ELISA and positive according to IgM ELISA (recent Lassa exposure).
[d] Ag−/IgM− = Samples testing negative according to both Ag and IgM ELISA.
[e] IgG serostatus = Immunoglobin G ELISA test result for detecting convalescent Lassa fever exposure.
[f] Calculated using Pearson's chi-square test assessing general differences in proportions between serostatus groups.

**Characteristic**	**Survival outcome**		**p value**[c]
	**Died (n = 106)**	**Discharged (n = 29)**	
Admission status			
Admitted	78 (74)	24 (83)	.308
Not admitted	28 (26)	5 (17)	
Gender			
Female	51 (49)	14 (48)	.978
Male	54 (51)	15 (52)	
Age, years			
< 5	17 (16)	8 (28)	
5-14	18 (17)	8 (28)	.178
15-40	58 (56)	12 (41)	
> 40	11 (11)	1 (3)	
District			
Bo	8 (9)	3 (10)	.806
Kenema	74 (80)	24 (83)	
Other	10 (11)	2 (7)	
Time since illness onset			
< 7 days	35 (46)	10 (38)	.501
≥ 7 days	41 (54)	16 (62)	
IgG serostatus[a]			
Positive	12 (12)	7 (25)	.101
Negative	85 (88)	21 (75)	
Season of presentation[b]			
Rainy	44 (42)	19 (66)	.022
Dry	62 (58)	10 (34)	

Table S2. Characteristics of confirmed Lassa fever cases by survival outcome, Kenema Government Hospital Lassa Fever Ward, 2012-2019. Note. All results expressed as frequencies and percentages unless indicated otherwise. Those characteristics with aggregate frequencies less than their respective aggregate serostatus group sample sizes

reflect missing characteristic data. Patient survival outcome was determined at hospital discharge (or following initial consultation for non-hospitalized subjects).
[a] IgG serostatus = Immunoglobin G ELISA test result for detecting convalescent Lassa fever exposure.
[b] Rainy and dry seasons were defined as May 1-October 31 and November 1-April 30, respectively.
[c] Calculated using Pearson's chi-square test assessing general differences in proportions between serostatus groups.

Serostatus group / Admission status	Survival outcome		p value[d]
	Died (n = 432)	Discharged (n = 400)	
Overall			<.001
Admitted	146 (34)	217 (54)	
Not admitted	286 (66)	183 (46)	
Ag+[a]			.308
Admitted	78 (74)	24 (83)	
Not admitted	28 (26)	5 (17)	
Ag−/IgM+[b]			<.001
Admitted	31 (46)	116 (78)	
Not admitted	36 (54)	33 (22)	
Ag−/IgM−[c]			<.001
Admitted	37 (14)	77 (35)	
Not admitted	222 (86)	145 (65)	

Table S3. Hospital admission status by survival outcome, Kenema Government Hospital Lassa Fever Ward, 2012-2019. Note. All results expressed as frequencies and percentages unless indicated otherwise. Those characteristics with aggregate frequencies less than their respective aggregate serostatus group sample sizes reflect missing characteristic data. Patient survival outcome was determined at hospital discharge (or following initial consultation for non-hospitalized subjects).
[a] Ag+ = Samples testing positive according to Ag ELISA (acute Lassa exposure).
[b] Ag−/IgM+ = Samples testing negative according to Ag ELISA and positive according to IgM ELISA (recent Lassa exposure).
[c] Ag−/IgM− = Samples testing negative according to both Ag and IgM ELISA.
[d] Calculated using Pearson's chi-square test assessing general differences in proportions between serostatus groups.

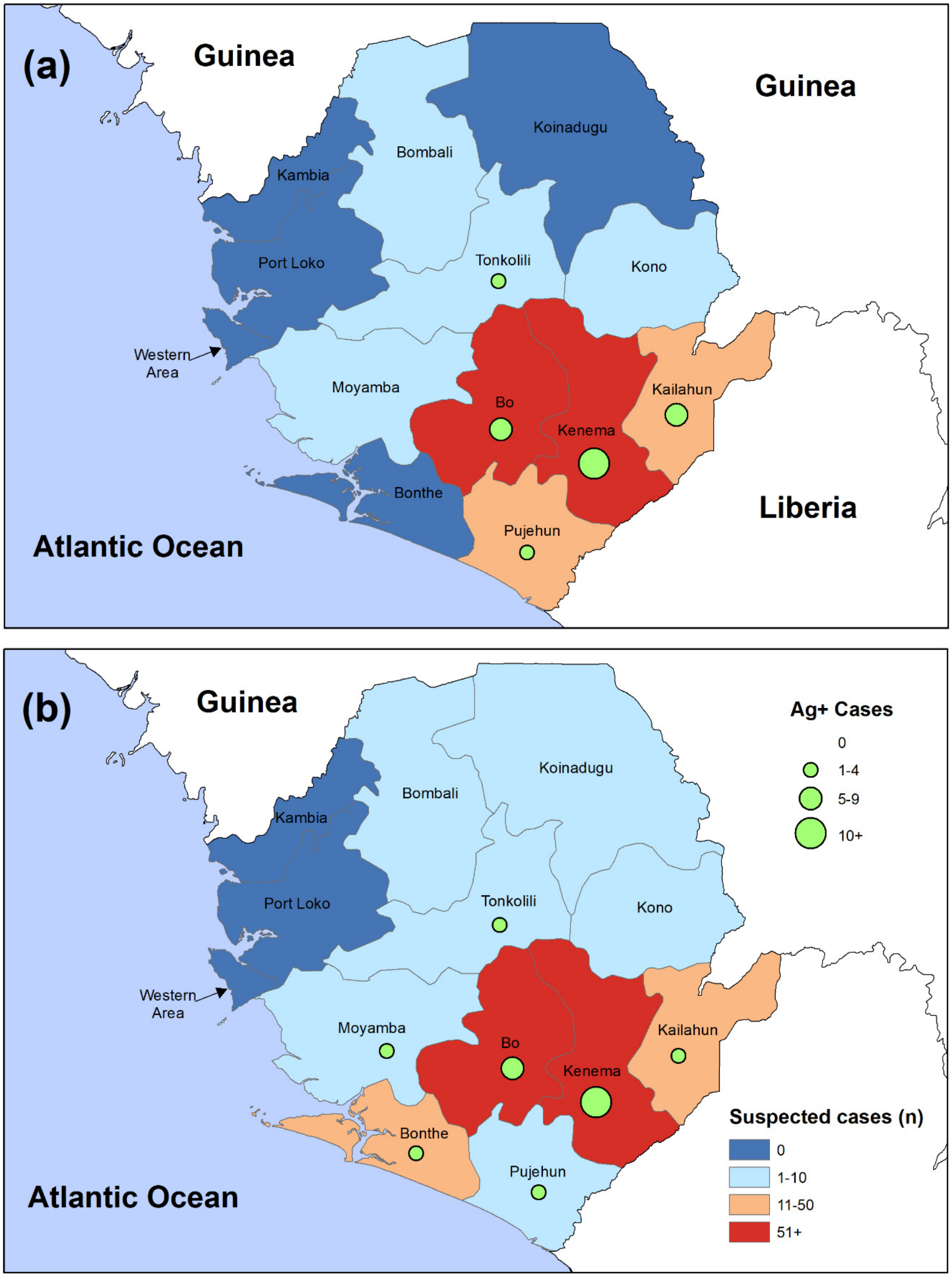

Figure S1. Spatial distribution of Lassa fever screenings and confirmed LF cases at Kenema Government Hospital by season of clinical presentation, 2012-2019. (a) Screenings during the rainy season (May 1 to October 31); (b) Screenings during the dry season (November 1 to April 30).
